# Relation between Resting State Front-Parietal EEG Coherence and Executive Function in Parkinson's Disease

**DOI:** 10.1155/2016/2845754

**Published:** 2016-06-28

**Authors:** Hiroko Teramoto, Akihiko Morita, Satoko Ninomiya, Takayoshi Akimoto, Hiroshi Shiota, Satoshi Kamei

**Affiliations:** Division of Neurology, Department of Medicine, Nihon University School of Medicine, 30-1 Oyaguchi Kami-cho, Itabashi-ku, Tokyo 173-8610, Japan

## Abstract

*Objective*. To assess the relation between executive dysfunction (ED) in Parkinson's disease (PD) and resting state functional connectivity evaluated using electroencephalography (EEG) coherence.* Methods*. Sixty-eight nondemented sporadic PD patients were assessed using the Behavioural Assessment of the Dysexecutive Syndrome (BADS) to evaluate executive function. EEG coherence in the left frontoparietal electrode pair (F3-P3) and the right frontoparietal electrode pair (F4-P4) was analyzed in the alpha and theta range. The BADS scores were compared across the coherence groups, and the multiple logistic regression analysis was performed to assess the contribution of confounders.* Results*. The standardized BADS score was significantly lower in the low F3-P3 coherence group in the alpha range (Mann-Whitney *U* test, *p* = 0.032), though there was no difference between F4-P4 coherence group in the alpha range, F3-P3, and F4-P4 coherence groups in the theta range and the standardized BADS score. The multiple logistic regression analysis revealed the significant relation between the F3-P3 coherence group in alpha range and age-controlled standardized BADS score (*p* = 0.039, 95% CI = 1.002–1.062).* Conclusion*. The decrease in resting state functional connectivity between the frontal and parietal cortices especially in the left side is related to ED in PD.

## 1. Introduction

Cognitive impairment is a common symptom of Parkinson's disease (PD) [1], and executive dysfunction (ED) is a well-known cognitive impairment in PD [2]. Executive function refers to a set of cognitive processes that control goal-directed behaviors, from goal formulation and intention formation to successful execution and processing of the outcome [3]. ED is a nonmotor symptom of PD and presents in early and late stages of the disease [3]. ED in PD has a negative impact on patients' quality of life and affects caregiver burden [3, 4]. ED in PD has been studied with many neuropsychological assessments including the Wisconsin Card Sorting Test, Trail Making Test [3], and the Behavioral Assessment of the Dysexecutive Syndrome (BADS) [5]. ED is characterized by deficits in internal control of attention, set shifting, planning, inhibition, conflict resolution, impairment in dual-task performance, and a range of decision-making and social cognition tasks [3].

The underlying mechanism of ED in PD is not clearly understood. Executive function is multifaceted; frontal and parietal cortical regions are reciprocally interconnected with each other and to the basal ganglia and thalamus in executive function [6]. Some studies have suggested that ED in patients with PD is caused by degeneration of the basal ganglia and/or frontal cortex [7, 8]. Neuroimaging studies using task-related fMRI have suggested that executive function in patients with PD is associated with fronto-parietal-striatal networks [6, 9]. Recently, the relation between the functional connectivity of resting state networks and cognitive impairment has been investigated. Tessitore et al. recently found that the default mode network (DMN) was different in 16 cognitively normal PD patients as compared to 16 healthy controls and, in PD, decreases in functional connectivity were located in the temporoparietal cortex within the DMN [10, 11]. Van Eimeren et al. revealed changes specifically in the posterior node of the DMN in seven unmedicated PD patients as compared to seven healthy controls [10, 12]. In PD, a limited number of studies have assessed the relation between resting state functional connectivity and executive function.

EEG coherence is a fundamental hallmark of integrated cortical functions [13]. EEG coherence is often used to assess functional connectivity in the human cortex [14]. A previous study reported that, in a resting state, patients with PD had lower EEG coherence within parietal electrodes at around 10 Hz than healthy controls [13].

In order to disclose changes in functional connectivity in PD patients with ED, this study assessed the relation between ED and EEG coherence in frontal and parietal regions in nondemented patients with PD.

## 2. Patients and Methods

### 2.1. Patients

Patients with sporadic PD were consecutively enrolled at the Neurology Clinic of Nihon University Itabashi Hospital between December 2006 and October 2008. The clinical diagnosis of sporadic PD was made according to the UK PD Brain Bank criteria [15]. Patients with other forms of Parkinsonism including drug-induced Parkinsonism, vascular Parkinsonism, dementia with Lewy bodies [16, 17], and atypical Parkinsonism with absent or minimal responses to anti-Parkinsonian drugs were excluded. Dementia with Lewy bodies was defined as the onset of dementia within 1 year of the onset of motor symptoms and did not have a history of visual hallucinations. Cranial magnetic resonance images were obtained from all patients, and patients with ischemic changes including a single lacuna and/or slight periventricular hyperintensity on T2-weighted images and fluid-attenuated inversion recovery images [18] were excluded. Patients who were given drugs that may influence EEG such as antianxiety drugs and psychotropic drugs were excluded.

All patients were assessed using the Mini-Mental State Examination (MMSE) based on the* Diagnostic and Statistical Manual of Mental Disorders, 4th edition *(DSM-IV) criteria for dementia, in accordance with a previous study [19]. Patients with an MMSE score <24 were excluded.

Executive function was assessed using the BADS [20]. The BADS is composed of six subtests that evaluate various aspects of ED and includes assessment for ED in a wide range of daily activities with ecological validity and is sensitive to ED in patients with PD [2, 5]. ED was defined as an age-controlled standardized score <70.

Written informed consent for participation in the present study was obtained from each patient according to a protocol approved by the Institutional Research Review Board of Nihon University.

### 2.2. Assessment of EEG Coherence

EEG examinations were performed using a magnetic optical disk with 16 electrode locations according to the International 10/20 System using a digital EEG instrument (Neurofax EEG-1100; Nihon Kohden, Tokyo, Japan). The EEG channels were recorded with a sampling rate of 200 Hz. EEG data were obtained with the patient in a resting awake condition with eyes closed. EEG coherence in the left frontoparietal electrode pair (F3-P3) and the right frontoparietal electrode pair (F4-P4) was analyzed using the EEG Analysis Software EMSE® Suite version 5.5.2 (SSI Inc., La Mesa, CA). Waves with a frequency from 1 Hz to 31 Hz were analyzed with a bandpass filter. The analysis window duration was 1 s with 80% overlapping. The data were divided into four frequency bands: delta (1.56–3.12 Hz), theta (3.90–7.80 Hz), alpha (8.59–12.48 Hz), and beta (13.26–29.64 Hz). Coherence was calculated in the alpha and theta range. Patients were divided into two groups based on the EEG coherence: low coherence (coherence <0.5) and high coherence (coherence ≥0.5).

### 2.3. Statistical Analysis

IBM SPSS statistics for Windows version 22 (IBM Corp., Armonk, NY) was used for statistical analysis. The Shapiro-Wilk normality test was used to evaluate whether continuous variables exhibited a normal distribution and nonparametric analysis was used for all nonnormal data. Demographic, clinical, and neuropsychological variables were compared across the coherence groups. Patient age was classified as ≤50 years, 51–60 years, 61–70 years, or >70 years. Patient age, sex, and Hoehn and Yahr (HY) stage were compared across the two coherence groups using Fisher's exact test. Disease duration, standardized BADS score, and age-controlled standardized BADS score were compared across the coherence groups using the Mann-Whitney *U* test. The relation between coherence group (low, high) and ED (present, absent) was assessed by Fisher's exact test. When the significant relation between the two coherence groups and standardized BADS score and/or age-controlled standardized BADS score was found, multiple logistic regression analysis was done to assess the contribution of confounders (age distribution, sex, disease duration, and distribution of HY stage). The level of statistical significance was defined as 0.05 for all tests.

## 3. Results

Eighty-nine patients with PD were enrolled and analyzed EEG coherence. However, 21 patients with MMSE score below 24 points were excluded. Characteristics of the 68 patients with MMSE score over 24 points are shown in Table 1. Median HY stage for all enrolled patients was 3.0.

Table 2 shows the relation between coherence and ED in PD patients. In alpha range, there was a significant difference in the distribution of PD patients with ED between high F3-P3 coherence group and low F3-P3 coherence group (Fisher's exact test, *p* = 0.016). There was a significant difference in the distribution of PD patients with ED between high F4-P4 coherence group and low F4-P4 coherence group (Fisher's exact test, *p* = 0.041). ED was significantly more frequent in low coherence group in alpha range. In theta range, there were no significant differences in the distribution of PD patients with ED between high coherence group and low coherence group.

Table 3 shows relation between EEG alpha coherence in the frontoparietal electrode pair and clinical characteristics in PD patients. Age distribution, sex, distribution of HY stage, and disease duration were not significantly different between high F3-P3 coherence group and low F3-P3 coherence group. The standardized BADS score was significantly different between high F3-P3 coherence group and low F3-P3 coherence group (Mann-Whitney *U* test, *p* = 0.032). The standardized BADS score was lower in the low F3-P3 coherence group. On the other hand, the standardized BADS score was not significantly different between high F4-P4 coherence group and low F4-P4 coherence group (Mann-Whitney *U* test, *p* = 0.055).

The multiple logistic regression analysis revealed the significant relation between the F3-P3 coherence group in alpha range and age-controlled standardized BADS score (*p* = 0.039, odds ratio = 1.03, 95% CI = 1.002–1.062). Other factors, including patients' age distribution, sex, disease duration, and distribution of HY stage, were not significant (Table 4). The relation between the F4-P4 coherence group in alpha range and the standardized BADS score using the multiple logistic regression analysis was not statistically significant (*p* = 0.053, 95% CI = 0.9997–1.057).

## 4. Discussion

We assessed the relation between ED and resting state EEG coherence in the alpha and theta frequency range in nondemented patients with PD. Our results showed a difference in EEG coherence in alpha range between nondemented PD patients with and without ED and no significant difference in theta range. EEG data were obtained with the patient in a resting awake condition with eyes closed and alpha wave recorded mainly in this state, in general. This might cause no significant difference of coherence between nondemented PD patients with and without ED in theta range. Low EEG coherence between the frontal and parietal region in alpha frequency range was associated with poor executive task performance. EEG is directly related to dynamic postsynaptic activity in the cortex [21] and has a wide spectrum of clinical applications [22]. A previous study reported that, in a resting state, patients with PD had lower EEG coherence within parietal electrodes at around 10 Hz than healthy controls [13]. Another study reported differences in the resting state EEG coherence pattern in the alpha frequency range between healthy subjects and patients with Alzheimer's disease, PD, and PD with dementia [22]. The frequency of the patients with ED was significantly higher in low coherence group in alpha, not only on the left side but also on the right side; however, the multiple logistic regression analysis revealed the significant relation between coherence in alpha range and age-controlled standardized BADS score only in the left side. Relation between ED and laterality of functional connectivity in PD was unclear. Rektorova reported significant correlation between functional connectivity of bilateral inferior parietal cortex within DMN and attention/executive function in PD patients [10]. Baggio et al. reported reduction of connectivity between the dorsal attention network and right frontoinsular regions associated with worse performance in attention/executive functions in mild cognitive impairment PD patients [23]. Our result suggested ED in PD is associated with a reduction of resting state functional connectivity in the frontal and parietal cortices, especially on the left side.

fMRI is one of the tools used to assess resting state functional connectivity and can be used to detect coherent fluctuations of blood-oxygenation-level-dependent signals [24, 25]. An fMRI study suggested that, in healthy subjects, executive task performance correlated with resting state lateral parietal nodes in a network that links the dorsolateral frontal and parietal cortices [25]. It has been suggested that the topological properties of brain networks are altered in PD patients with mild cognitive impairment, including dysfunction of executive function, attention, visuospatial functions, and memory. These findings have been obtained using graph-theoretical analyses of functional networks obtained with resting state fMRI [23]. In PD patients who were cognitively unimpaired, the decreased DMN connectivity significantly correlated with cognitive parameters but not with disease duration, motor impairment, or levodopa therapy [11].

The fMRI signal is only an indirect measure of neuronal activity [26], and fMRI is expensive to operate. The time resolution of EEG is in the range of milliseconds [27]. In addition, EEG is of low cost and represents no risk to the patient [22]. EEG coherence is therefore a good tool with which to assess changes in functional connectivity in PD.

This study revealed that low EEG coherence between the left frontal and left parietal region was associated with poor executive task performance in PD. A decrease in resting state functional connectivity between the frontal and parietal cortices is related to ED in PD.

## Figures and Tables

**Table 1:** Clinical features.

Number of patients	68

Age (years)	68.0 (34–87)
≤50	6
51–60	15
61–70	22
>70	25

Sex	
Male	35
Female	33

Disease duration (months)	60 (9–228)

HY stage	3.0 (1–5)
I	4
II	31
III	28
IV	4
V	1

Standardized BADS score	87.5 (46–124)

Age-controlled standardized BADS score	93 (53–124)

Data are expressed as median (range) or *n*.

BADS: Behavioral Assessment of the Dysexecutive Syndrome; HY stage: Hoehn and Yahr stage.

**Table 2:** Relation between EEG coherence in the frontoparietal electrode pair and ED in PD patients.

Range		Age-controlled standardized BADS score		*p*
		<70 (patients with ED)	≥70 (patients without ED)	
Alpha	Low F3-P3 (left side) coherence group	13	26	0.016^*∗*^
	High F3-P3 (left side) coherence group	2	27	
	Low F4-P4 (right side) coherence group	11	22	0.041^*∗*^
	High F4-P4 (right side) coherence group	4	31	

Theta	Low F3-P3 (left side) coherence group	12	38	0.742
	High F3-P3 (left side) coherence group	3	15	
	Low F4-P4 (right side) coherence group	8	32	0.768
	High F4-P4 (right side) coherence group	7	21	

Data are expressed as number of patients.

BADS: Behavioral Assessment of the Dysexecutive Syndrome; ED: executive dysfunction; PD: Parkinson's disease.

ED was defined as age-controlled standardized BADS score <70.

The low coherence group had coherence <0.5 and the high coherence group had coherence ≥0.5.

The relation between two coherence groups and ED was assessed using Fisher's exact test. ^*∗*^Statistically significant (*p* < 0.05).

**Table 3:** Relation between EEG alpha coherence in the frontoparietal electrode pair and clinical characteristics in PD patients.

	Low F3-P3 (left side) coherence group	High F3-P3 (left side) coherence group	*p*	Low F4-P4 (right side) coherence group	High F4-P4 (right side) coherence group	*p*
Age (years)	70.0 (42–87)	61.5 (34–81)		70.5 (42–87)	59.0 (34–76)	
≤50	2	4	0.144	1	5	0.002^*∗*^
51–60	7	10		3	14	
61–70	13	9		13	9	
>70	18	7		17	8	

Sex						
Male	23	14	0.469	18	19	1.00
Female	17	16		16	17	

Disease duration (months)	48 (9–192)	60 (9–228)	0.130	50 (9–192)	60 (9–228)	0.300

HY stage	3.0 (1–5)	2.0 (1–4)				
I	3	2	0.464	1	4	0.012^*∗*^
II	15	16		12	19	
III	19	9		20	8	
IV	2	3		1	4	
V	1	0		0	1	

Standardized BADS score	85 (46–114)	95 (56–124)	0.032^*∗*^	85 (51–114)	95 (46–124)	0.055

Age-controlled standardized BADS score	89 (54–124)	94 (53–124)	0.063	84 (59–124)	94 (53–124)	0.152

Data are expressed as median (range) or *n*.

BADS: Behavioral Assessment of the Dysexecutive Syndrome; HY stage: Hoehn and Yahr stage; PD: Parkinson's disease.

The low coherence group had coherence <0.5 and the high coherence group had coherence ≥0.5.

Age, sex, and HY stage were compared across groups using Fisher's exact test. Disease duration, standardized BADS score, and age-controlled standardized BADS score were compared across groups using the Mann-Whitney *U* test. ^*∗*^Statistically significant (*p* < 0.05).

**Table 4:** Effect of confounders on prevalence between EEG alpha coherence in the left frontoparietal electrode pair and age-controlled standardized BADS score in PD patients by multiple logistic regression analysis.

	*B*	SE	*χ* ^2^	*p*	Odds ratio	95% CI
Age						
≤50 years = 1	−0.42	0.30	1.86	0.17	0.66	0.36–1.20
51 to 60 years = 2						
61 to 70 years = 3						
>70 years = 4						
Sex						
Male = 1, female = 0	−1.03	0.56	3.45	0.063	0.36	0.12–1.06
Disease duration						
Month, real number	0.0040	0.0057	0.50	0.48	1.00	0.99–1.02
HY stage						
Real number	−0.24	0.43	0.31	0.58	0.79	0.34–1.82
Age-controlled standardized BADS score						
Real number	0.031	0.015	4.25	0.039^*∗*^	1.03	1.002–1.062

A dichotomous dependent variable of EEG alpha coherence in the left frontoparietal electrode pair was assigned a value of 0 when coherence was <0.5 and 1 when coherence was ≥0.5.

BADS: Behavioral Assessment of the Dysexecutive Syndrome; HY stage: Hoehn and Yahr stage; *B*: regression coefficient; SE: standard error; 95% CI; 95% confidence interval.

^*∗*^Statistically significant (*p* < 0.05).
